# The role of multi-organ cancer predisposition genes in **the risk of** inherited and histologically diverse gastric cancer

**DOI:** 10.1016/j.ebiom.2025.105759

**Published:** 2025-05-29

**Authors:** Joana Guerra, Ana P. Estrada-Florez, Paul C. Lott, Carla Pinto, Manuela Pinheiro, Katherine A. Chiu, Dennis J. Montoya, Hongyong Zhang, Guadalupe M. Polanco-Echeverry, Pedro Pinto, Ana Peixoto, Catarina Santos, Ana Barbosa, João Silva, John Suarez-Olaya, Fabian Castro-Valencia, Graciela Molina, Alejandro H. Corvalán, Adriana Della Valle, Jose E. Castelao, Nereida Fernandez-Fernandez, Lucia Cid, Nora Rios-Sarabia, Rafael Medrano, Alejandra Mantilla, Maria M. Echeverry de Polanco, Ana L. Rivera-Herrera, Julián Riaño-Moreno, Rafael Parra-Medina, Luz M. González-Castrillón, Ricardo Dominguez, Ana R. Isidoro, Fernanda Silva, Douglas R. Morgan, Alicia M. Cock-Rada, Maria C. Sanabria-Salas, Mabel H. Bohorquez, Javier Torres, Manuel R. Teixeira, Luis G. Carvajal-Carmona, Consorcio Galiciaab, Hernandez Vicent, Alonso Sara, Galovart Miguel, Rodriguez-D'Jesus Antonio, de Castro Luisa, Rodriguez-Prada José Ignacio, Gago-Domínguez Manuela, Redondo-Marey Carmen, Miranda Ponte Sara, The Hispanic Gastric Cancer Genetics Collaborative Groupac, González Patricio, Parra Carol, Torres Osvaldo, Adelsdorfer Cedric, Martínez Jose, Norwood Dalton, Montalvan-Sanchez Eleazar, Nefa Florencia, Sanchez Gloria, Castaño Rodrigo, García Francisco, Buitrago Diego Andrés

**Affiliations:** a Cancer Genetics Group, IPO-Porto Research Center (CI-IPOP)/RISE@CI-IPOP (Health Research Network), Portuguese Oncology Institute of Porto (IPO-Porto)/Porto Comprehensive Cancer Center, Porto, Portugal; b Doctoral Programme in Biomedical Sciences, School Medicine and Biomedical Sciences, University of Porto (ICBAS-UP), Porto, Portugal; c Department of Laboratory Genetics, Portuguese Oncology Institute of Porto (IPO-Porto)/Porto Comprehensive Cancer Center, Porto, Portugal; d The Health Equity Leadership, Science, and Community Research Laboratory, Genome Center, University of California, Davis, USA; e Grupo de Citogenética, Filogenia y Evolución de Poblaciones, Facultades de Ciencias y Facultad de Ciencias de la Salud, Universidad del Tolima, Ibagué, Colombia; f Department of Pathological, Cytological and Thanatological Anatomy, School of Health, Polytechnic Institute of Porto, Porto, Portugal; g Escuela de Medicina, Facultad de Ciencias de la Salud, Universidad Autónoma de Chile, Providencia, Chile; h Departamento de Hematología y Oncología, Facultad de Medicina, Pontificia Universidad Católica de Chile, Santiago, Chile; i Advanced Center for Chronic Diseases, Santiago, Chile; j Grupo Colaborativo Uruguayo, Investigación de Afecciones Oncologicas Hereditarias, Montevideo, Uruguay; k Oncology and Genetics Unit, Instituto de Investigacion Sanitaria Galicia Sur (IISGS), Xerencia de Xestion Integrada de Vigo-SERGAS, Vigo, Spain; l Department of Gastroenterology, Complexo Hospitalario Universitario de Vigo (CHUVI), SERGAS, Vigo, Spain; m Research Group in Digestive Diseases, Galicia Sur Health Research Institute (IIS Galicia Sur), SERGAS-UVIGO, Vigo, Spain; n Unidad de Investigación en Enfermedades Infecciosas y Parasitarias, Unidad Médica de Alta Especialidad en Pediatría, Instituto Mexicano del Seguro Social, México City, Mexico; o Dirección general, Unidad Medica de Alta Especialidad en Oncología Instituto Mexicano del Seguro Social (IMSS), México City, Mexico; p Grupo de Investigación en Biología del Cáncer, Instituto Nacional de Cancerología, Bogotá, Colombia; q Department of Pathology, Instituto Nacional de Cancerología, Bogotá, Colombia; r Facultad de Medicina, Universidad Cooperativa de Colombia, Villavicencio, Colombia; s Research Institute, Fundación Universitaria de Ciencias de la Salud - FUCS, Bogotá, Colombia; t Facultad de Medicina, Universidad de Antioquia, Medellín, Colombia; u Hospital de Occidente, Ministry of Health, Santa Rosa de Copan, Copan, Honduras; v Department of Pathology, Portuguese Oncology Institute of Porto (IPO Porto)/Porto Comprehensive Cancer Center, Porto, Portugal; w UAB Division of Gastroenterology and Hepatology, The University of Alabama at Birmingham, Birmingham, AL, USA; x Instituto de Cancerologia Las Américas Auna, Medellín, Colombia; y School of Medicine and Biomedical Sciences (ICBAS), University of Porto, Porto, Portugal; z European Reference Network on Genetic Tumour Risk Syndromes (ERN GENTURIS), Porto, Portugal; aa Department of Biochemistry and Molecular Medicine, School of Medicine, University of California, Davis, CA, USA

**Keywords:** Stomach cancer, Gastric cancer, Familial aggregation, Genetic predisposition, Homologous recombination, Germline variants

## Abstract

**Background:**

Approximately 10% of cases with gastric cancer (GC) exhibit familial clustering, however, only 1–3% of cases can be explained by two known hereditary syndromes: Hereditary Diffuse Gastric Cancer (HDGC) caused by *CDH1* and *CTNNA1* pathogenic germline variants; and Gastric Adenocarcinoma and Proximal Polyposis of the Stomach (GAPPS), caused by germline variants in *APC* 1B promoter. Familial intestinal gastric cancer (FIGC) has been defined clinically, but it remains mostly genetically unexplained. Likewise, the heritability of mixed histology GC remains to be known. We aimed to estimate the frequency of known cancer predisposition gene variants in GC cases with and without a cancer family history, diverse histological subtypes, and varied age of onset.

**Methods:**

We evaluated the contribution of pathogenic or likely pathogenic (P/LP) variants in well-established moderate-to-high penetrance multi-organ cancer predisposition genes for GC risk in a large international multi-centre retrospective cohort study involving 750 patients with GC of early-onset or family history of cancer, either by panel sequencing or whole exome sequencing (WES). Panel sequencing was conducted on 328 cases, while WES was performed on the remaining 422. Tumour sequence analyses were performed on samples from 15 patients with P/LP variants. Mutations identified in five index cases were also tested in their relatives.

**Findings:**

We identified 45 patients (6%) with P/LP variants in: *ATM* (17 cases), *BRCA2* (10 cases), *MLH1* (five cases), *TP53* (three cases), *BRCA1*, *PALB2*, *RAD51D*, and *CHEK2* (two patients each), and *RAD51C* and *PMS2* (one case each), all of which were mutually exclusive. The P/LP variant prevalence was higher in intestinal (9.8%) than in diffuse (4.3%) or mixed GC (4.5%) (*p-value* = 0.023), without difference per mutated gene by histological subtypes. Only 16 of the 45 patients who carried P/LP variants fulfilled the National Comprehensive Cancer Network genetic testing criteria of at least one cancer predisposition syndrome.

**Interpretation:**

Our findings indicate that a broader panel of cancer predisposition genes, beyond *CDH1* and *CTNNA1*, should be included in gene panels to investigate germline variants in patients with GC. This would be especially beneficial when there is a family history of cancer, irrespective of histology subtype, as it would increase the chance of identifying patients who could benefit from risk reduction, targeted treatment, and surveillance of other cancer types.

**Funding:**

10.13039/100000054National Cancer Institute of the National Institutes of Health, USA; 10.13039/100019348Universidad del Tolima, Colombia; 10.13039/100022965MINCIENCIAS, Colombia; L'OREAL-UNESCO-ICETEX-COLCIENCIAS, Colombia; 10.13039/100019346Instituto Nacional de Cancerología, Colombia; 10.13039/100000043American Association for Cancer Research, USA; 10.13039/100020266ANID Ministerio de Ciencia, Chile; 10.13039/501100002850Fondecyt, Chile; 10.13039/501100002848CONICYT/10.13039/501100018735ANID FONDAP, Chile; 10.13039/501100004881Instituto Mexicano del Seguro Social and 10.13039/501100003141Consejo Nacional de Ciencia y Tecnología, México; IPO Porto, Portugal; 10.13039/501100011733Liga Portuguesa Contra o Cancro, Portugal; Fundacao para a Ciencia e Tecnologia, Portugal; The Auburn Community Cancer Endowed Chair in Basic Research, USA; The Heart, BrEast, and BrAin HeaLth Equity Research (HEAL HER) program, a program made possible by residual class settlement funds in the matter of *April Krueger v. Wyeth, Inc*., Case No. 03-cv-2496 (US District Court, SD of Calif.), USA.

**Research in context**

**Evidence before this study**

Gastric cancer (GC) is the third most commonly diagnosed cancer worldwide and poses a significant burden, particularly in Asia and Latin America. Most GC etiological studies have concentrated on lifestyle and environmental risk factors, while relatively few addressing genetic risk have been conducted in non-White/European populations. Approximately 10% of patients with GC report a family history of the disease, but only about 1–3% are linked to known hereditary syndromes. The genetics of GC in families of patients with non-diffuse histology gastric tumours (intestinal or mixed) remains poorly understood.

**Added value of this study**

This study investigated the genetics of GC in a large, international, ethnically diverse, multi-centre cohort that included a significant proportion of Latin American patients. We demonstrated that known moderate-to-high cancer genes could account for a substantial percentage of GC families, regardless of histological type.

**Implications of all the available evidence**

The findings suggest that gene panels used to investigate germline variants in patients with GC should include multi-organ cancer predisposition genes, especially in those with a family history of cancer and early onset, regardless of tumour histology, due to the significant overlap in characteristics of various cancer predisposition syndromes. The high prevalence of homologous recombination gene variants is also clinically relevant, as patients who carry germline variants may benefit from targeted therapies such as PARP inhibitors.

## Introduction

Gastric cancer (GC) ranks fifth in both incidence and cancer-related deaths worldwide.^1^^,^^2^ The disparity between GC incidence and mortality rates highlights its poor prognosis. Only a small fraction of cases are detected at early stages, resulting in poor outcomes for patients.^3^^,^^4^ GC familial clustering occurs in ∼10% of the cases; however, only 1–3% of GCs result from a known hereditary syndrome.^5^ The most well-known GC inherited form is Hereditary Diffuse Gastric Cancer (HDGC), a syndrome associated with the presence of diffuse-type histology gastric carcinomas caused by germline variants in *CDH1* or *CTNNA1*.^1^^,^^6^ Nonetheless, pathogenic/likely pathogenic (P/LP) variants in these two genes only account for ∼42% of HDGC families.^7^^,^^8^ In 2016, the genetic basis of Gastric Adenocarcinoma and Proximal Polyposis of the Stomach (GAPPS) Syndrome, was elucidated in a handful of families known to carry germline variants in *APC* 1B promoter.^9^^,^^10^ While most cases of Familial Intestinal Gastric Cancer (FIGC) are known to follow an autosomal dominant inheritance pattern,^11^ FIGC's genetic background is not fully understood. Similarly, mixed histology GC is not usually considered as being within the HDGC tumour spectrum,^12^ raising questions about the heritability of mixed gastric cancer.

We and others have identified families and patients with GC harbouring germline variants in multi-organ cancer genes such as *ATM*, *BRCA1*, *BRCA2*, and *PALB2*, suggesting that these genes may be involved in GC predisposition.^13^, ^14^, ^15^, ^16^, ^17^, ^18^ As genes involved in homologous recombination (HR) repair, these findings are of dual importance: genetic risk information can be utilised in genetic counselling, surveillance, and risk reduction measures, while also contributing to the development of novel GC treatments. HDR-deficient tumours may be effectively treated with PARP inhibitors (PARPi,^19^, ^20^, ^21^). Despite these recent findings, there remain significant knowledge gaps regarding the role and importance of HDR genes and other multi-organ cancer genes in GC risk, including their mutation prevalence in European and non-European populations, their impact on the pathogenesis of different GC histological subtypes, and their evidence of GC causality. To address some of the knowledge gaps, in this study we investigated the prevalence of P/LP germline variants in the multi-organ moderate-to-high penetrance cancer predisposition genes *ATM*, *BRCA2*, *BRCA1*, *PALB2*, *TP53*, *RAD51D*, *RAD51C*, *CHEK2*, *MLH1*, *MSH2*, *MSH6*, and *PMS2* in 750 patients with GC (with early-onset and/or a family history of cancer), recruited in a multi-centre study of several high-risk countries in Latin America and Europe, such as Portugal, Spain, Colombia, and Mexico.^2^ We found that 6% of these patients carried P/LP variants, with a high prevalence of P/LP variants among those with intestinal tumours, with approximately 1 in every 10 patients carrying a P/LP variant. Our study also found that only about a third of these patients would have been identified using current NCCN guidelines. Furthermore, our co-segregation and tumour analyses provided some evidence of GC causality in a subset of the mutation carriers. Therefore, these findings advance our knowledge of GC genetic predisposition and contribute to increasing our understanding of GC genetics in understudied populations in Latin America and provide further evidence for a role of multi-organ cancer genes in the risk of GC.

## Methods

### Ethics

We conducted the study in accordance with the Declaration of Helsinki. The participating institutions in the study that consented and recruited patients using locally approved research protocols (with approval numbers shown in brackets), included: 1) Colombia: Universidad del Tolima [2.3-128/07312018; 2.3-807/151111], Hospital Federico Lleras Acosta [7727/091411], Hospital Hernando Moncaleano [03/06/2011], Universidad de Antioquia [013/092613], Clínica Las Americas Auna [Acta 12/2015], and Instituto Nacional de Cancerología (INC) [CEI-01534-2]; 2) Honduras: Hospital de Occidente [05/12/2023]; 3) Mexico: Instituto Mexicano del Seguro Social [R-2013-785-045]; 4) Chile: Hospital Dr. Gustavo Fricke y Servicio de Salud de Viña del Mar-Quillota [23/2019], Servicio de Salud de Valparaíso-San Antonio [056/2019]; 5) Uruguay: Grupo Colaborativo Uruguayo: Investigación de Afecciones Oncologicas Hereditarias [Nota No 07/CE/2019]; 6) Spain: Complejo Hospitalario Universitario de Vigo [2020/091]; 7) Portugal: Instituto Portugues de Oncologia (IPO Porto) [351R1/018]. All patients enrolled at these institutions provided written informed consent, following locally approved ethical protocols. Details of the studies that recruited participants, including institutional authorities and approval numbers for all research protocols used in patient recruitment, can be found in Supplementary Table S1.

### Study population

We conducted a retrospective cohort study, including 750 index patients with GC from three cohorts: i) The multi-centre Hispanic Gastric Cancer Genetics Collaborative Group (HGC^2^G), which includes incident patients recruited in hospital-based case-control studies in Chile, Colombia, Honduras, Mexico, Spain, and Uruguay; ii) IPO Porto and; iii) The Hereditary Cancer Program of the Colombian INC.^22^ Patients from IPO Porto and INC were referred for genetic cancer risk assessments at the largest cancer hospitals in Portugal and Colombia, respectively (Supplementary Table S1). Lauren's histology classification was used in all cases, and local pathologists reviewed available tumours and pathology.^23^ The inclusion criteria for our study were: i) patients diagnosed with GC of any histology by age 50 years (y) or ii) GC diagnosed of any histology after age 50 years and family history of any cancer in first- and/or second-degree relatives. All patients with diffuse GC were negative for *CDH1* and *CTNNA1* P/LP variants. Genomic DNA was extracted by local investigators from peripheral blood samples using various DNA extraction kits according to the manufacturer's instructions. Sex was self-reported and verified after sequencing by analysing the ratio of sequencing reads that map to the X and Y chromosomes. Using P/LP data, family history, age at diagnosis and histological information, we used the National Comprehensive Cancer Network (NCCN) criteria for diagnosing hereditary breast and ovarian cancer (HBOC), the Bethesda criteria for Lynch syndrome, and the Chompret criteria for Li-Fraumeni syndrome.

### Multigene panel testing

The 328 cases from IPO Porto and INC underwent panel sequencing. The IPO Porto cohort (258 cases) was analysed using the TruSight Cancer Panel (reference FC-121-0202, Illumina, Inc., USA), with library preparation performed according to the manufacturer's protocol and 2 × 150 bp PE (paired-end) sequencing carried out in the Illumina MiSeq platform. Bioinformatic analyses were performed as previously described.^24^ The INC cohort (70 cases) was analysed using the Canadian Consortia Inherited Cancer Panel (customised probe panel reference #20011891; Illumina, Inc.) with library preparation performed according to the manufacturer's protocol and 2 × 150 bp PE sequencing in the Illumina MiSeq platform. Variant calling and annotation were carried out with the SOPHiA's DDM platform, using the ILL1IC1G3_TSC algorithm (Sophia Genetics, Switzerland). Sequence analyses were carried out as described previously,^25^ using the platforms VarSome (free version)^26^ and PathoMAN^27^ for automated curation and reviewed by an onco-geneticist and a biologist trained in genetics for interpretation.

### Whole exome sequencing

Whole exome sequencing (WES) was performed in the remaining 422 HGC^2^G cases from Chile (n = 33), Colombia (n = 216), Honduras (n = 45), Mexico (n = 92), Spain (n = 29), and Uruguay (n = 7) using SureSelect Human All Exome V7 (Part number 5191-4029, Agilent Technologies, Santa Clara, CA, USA) and 150 PE reads with an Illumina's NovaSeq6000 (Illumina, Inc.). Each sample was sequenced at ∼100× raw depth (∼6 Gb per sample). Alignment and variant calling were performed using Illumina Dragen v4.2. Variant calls were re-calibrated using GATK v4.0 variant recalibration. Variants were annotated with Illumina Nirvana and Annovar.

Our study focuses on identifying P/LP variants in known moderate-to-high penetrance multi-organ cancer predisposition genes shared among the two different multigene panels and WES (Supplementary Table S2). The following genes were analysed in the three cohorts: The *ATM* (NM_000051.3), *BRCA2* (NM_000059.3), *BRCA1* (NM_007294.3), *PALB2* (NM_024675.3), *TP53* (NM_000546.5), *RAD51D* (NM_002878.3), *RAD51C* (NM_058216.2), *CHEK2* (NM_007194.3), *MLH1* (NM_000249.3), *MSH2* (NM_000251.2), *MSH6* (NM_000179.2), and *PMS2* (NM_000535.5). All variants with a variant allele frequency (VAF) ≤20%, minor allele frequency (MAF) >1%, and/or intronic variants at more than 12 bp away from exon-intron boundaries were excluded. For MAF filtering, data was obtained from the 1000 Genomes Project Phase 3 Hg38 (1KGP, https://www.internationalgenome.org), Genome Aggregation Database (gnomAD, v3.1.2 https://gnomad.broadinstitute.org), and Exome Aggregation Consortium (ExAC, release 1, https://exac.broadinstitute.org) databases.

### Tumour analysis

DNA extraction, from eight formalin-fixed paraffin-embedded (FFPE) tumour samples from Portugal, was performed using the Cobas® DNA Sample Preparation Kit (Reference 05985536190; Roche Diagnostics, Switzerland) according to the manufacturer's instructions. Libraries were prepared using the Illumina DNA Prep with Enrichment protocol (Reference 20025524; Illumina, Inc.) with Illumina's TruSight Hereditary Cancer panel. Library preparation was performed following the manufacturer's protocol and 2 × 150 bp PE sequencing in Illumina's NextSeq 550 platform. Bioinformatic analyses were performed using a previously validated pipeline using the NextGENe software (v2.4.2.2, Softgenetics, State College, PA, USA) with a cut-off VAF of ≤5%.^15^

Genomic DNA from samples of fresh-frozen tumours from Mexico (n = 3) and Colombia (n = 4) was isolated using the DNeasy Blood & tissue kit (Reference 69504; Qiagen). Local surgical pathologists verified all tumour biopsies and surgical specimens. We tested for microsatellite instability (MSI) using PCR with five primary microsatellite markers (BAT-25, BAT-26, D2S123, D5S346, and D17S250) of the Bethesda panel as previously described^28^ and fragment analysis performed in capillary electrophoresis with an ABI DS33 kit (Reference 4345833; Applied Biosystems). WES was performed using SureSelect Human All Exome V7 (Reference 5191-4029; Agilent Technologies, Santa Clara, CA, USA). Somatic analysis of tumours utilised the Illumina DRAGEN Bio-IT Platform (Illumina, Inc) version 4.2. In brief, samples were aligned to the hg38 genome and paired tumour-normal variant calling using the somatic mode of the DNA pipeline using default settings. Homologous recombination deficiency (HRD) scoring was also performed in WES data in the DRAGEN platform. MSI scoring was calculated with MSISensor-Pro^29^ on WES data. Annotations were performed by Nirvana (Illumina, Inc) and oncoplot visualisation by MAFtools v2.24.2.^30^

### Statistics

This study used a convenience sampling approach. To minimise potential sources of bias, inclusion and exclusion criteria were applied consistently across all samples and cohorts. Statistical analysis was carried out with R (v.4.3.3). The statistical significance of the associations was performed using Pearson's Chi–squared test if expected counts were >5; otherwise, Fisher's exact test was used. Analysis of variance (ANOVA) F-test was used to compare the mean between groups. *p-values* < 0.05 were considered statistically significant.

### Role of funders

The funders did not participate in the study design, data collection, analysis, interpretation, or report writing.

## Results

A total of 750 patients with GC were included in this study (366 women, 384 men, Table 1, Supplementary Tables S3 and S4). Diffuse histology was the most prominent (n = 376; 53%), followed by intestinal (n = 224; 31.5%), and mixed (n = 110; 15.5%). Forty cases had adenocarcinomas of unknown/unreported histology. The average age at diagnosis was 51.1 years (range: 19–86), with significant differences in age between histological types (*p-value* < 0.001 [F-test]), with diffuse tumours diagnosed earlier (mean age 47.0 years; range: 19 years–86 years) than intestinal (mean age 56.3 years; range: 27 years–86 years) and mixed (mean age 55.4 years; range: 24 years–82). Women with GC were significantly younger than men (49.7 years vs. 53 years; *p-value* < 0.001 [F-test]). 81.5% of the cases had a family history of any cancer (563 of 689), and 54.7% had a family history of GC (313 of 691). Additionally, in 507 cases with stage information, 44.2% had stage I–II tumours, and 55.8% had advanced tumours. A higher percentage of diffuse tumours was diagnosed with stages III–IV (63%, 157 of 246) compared to intestinal (41.7%, 63 of 151; *p-value* = 0.00002 [Pearson's Chi-squared test]) and mixed tumours (54.2%, 52 of 96; *p-value* = 0.109 [Pearson's Chi-squared test]).

**Table 1:** Clinicopathological characteristics of the patients enrolled in the study, which included GC cases from Instituto Nacional de Cancerología in Colombia (INC), the Portuguese Oncology Institute of Porto (IPO Porto), and the Hispanic Gastric Cancer Genetics Collaborative Group (HGC^2^G).

Variable	Cohorts (n, %)			Total
	INC	IPO Porto	HGC^2^G	
Number of patients	70 (9.3)	258 (34.4)	422 (56.3)	750 (100)
**Inclusion criteria**				
Age at diagnosis ≤50 years without family history of cancer in PDR and/or SDR	10 (14.3)	33 (12.8)	144 (34.1)	187 (24.9)
Age at diagnosis ≤50 years and with family history of cancer in FDR and/or SDR	36 (51.4)	96 (37.2)	73 (17.3)	205 (27.3)
Age at diagnosis >50 years, and with family history of cancer in FDR and/or SDR	24 (34.3)	129 (50)	205 (48.6)	358 (47.7)
**Sex**				
Female	46 (65.7)	133 (51.6)	187 (44.3)	366 (48.8)
Male	24 (34.3)	125 (48.4)	235 (55.7)	384 (51.2)
**Age**				
Average (SD)	46.5 (13.9)	50.5 (12.8)	52.3 (13.8)	51.1 (13.6)
**Country**				
Chile	–	–	33 (7.8)	33 (4.4)
Colombia	70 (100)	–	216 (51.2)	286 (38.1)
Honduras	–	–	45 (10.7)	45 (6)
Mexico	–	–	92 (21.8)	92 (12.3)
Portugal	–	258 (100)	–	258 (34.4)
Spain	–	–	29 (6.9)	29 (3.9)
Uruguay	–	–	7 (1.7)	7 (0.9)
**Race/ethnicity**				
Latino	70 (100)		393 (93.1)	463 (61.7)
White		258 (100)	29 (6.9)	287 (38.3)
**Family history of any cancer in FDR and/or SDR**				
No	10 (14.3)	33 (12.8)	85 (23.4)	128 (18.5)
Yes	60 (85.7)	225 (87.2)	278 (76.6)	563 (81.5)
No data	–	–	59	59
**Family history of GC in FDR and/or SDR**				
No	33 (47.1)	110 (42.6)	235 (64.7)	378 (54.7)
Yes	37 (52.9)	148 (57.4)	128 (35.3)	313 (45.3)
No data	–	–	59	59
**Histology**				
Diffuse	39 (60.9)	133 (51.6)	204 (52.6)	376 (53)
Intestinal	21 (32.8)	63 (24.4)	140 (36.1)	224 (31.5)
Mixed	4 (6.3)	62 (24)	44 (11.3)	110 (15.5)
Adenocarcinoma	6		34	40
**Stage**^a^				
I	7 (10.8)	67 (28)	49 (24.1)	123 (24.3)
II	15 (23.1)	35 (14.6)	51 (25.1)	101 (19.9)
III	20 (30.8)	74 (31)	77 (37.9)	171 (33.7)
IV	23 (35.4)	63 (26.4)	26 (12.8)	112 (22.1)
Unknown	5	19	219	243

FDR: First degree relatives, SDR: Second degree relatives, SD: Standard deviation, GC: Gastric cancer.

a American Joint Committee on Cancer (AJCC) Staging Manual Edition 8th.

### Patients with pathogenic/likely pathogenic (P/LP) germline variants

Despite some differences in coverage between WES and panel sequencing (with lower coverage for *CHEC2*, *RAD51C*, and *MSH2* in WES, Supplementary Table S2), we identified 45 patients (6%) with a P/LP germline variant in *ATM*, *BRCA2*, *BRCA1*, *PALB2*, *TP53*, *RAD51D*, *RAD51C*, *CHEK2*, *MLH1*, or *PMS2* (Table 2, Supplementary Tables S3–S5), all of which were mutually exclusive.

**Table 2:** Clinical characteristics of the germline pathogenic variant carriers.

Gene	HGVS coding	HGVS protein	Effect	Dataset–Country	Sample ID	Sex	Age of diagnosis	Histology	FH of GC
*ATM*	NM_000051.4:c.640del	NP_000042.3:p.(Ser214ProfsTer16)	Frameshift deletion	IPO-Porto–Portugal	EI_1166	F	57	Diffuse	N
	NM_000051.4:c.3171dup	NP_000042.3:p.(Trp1058MetfsTer5)	Frameshift insertion	HGC^2^G–Colombia	I_27122	F	48	Diffuse	Y
	NM_000051.4:c.3381_3384del	NP_000042.3:p.(Gln1128MetfsTer5)	Frameshift deletion	HGC^2^G–Chile	I_27254	M	40	Diffuse	Y
	NM_000051.4:c.3802del	NP_000042.3:p.(Val1268Ter)	Nonsense	IPO-Porto–Portugal	EI_1110	F	32	Intestinal	N
	NM_000051.4:c.3802del	NP_000042.3:p.(Val1268Ter)	Nonsense	HGC^2^G–Colombia	I_27532	F	41	Diffuse	N
	NM_000051.4:c.4110-1G>C	NP_000042.3:p.?	Splice acceptor	HGC^2^G–Colombia	I_27102	M	56	Intestinal	Y
	NM_000051.4:c.4852C>T	NP_000042.3:p.(Arg1618Ter)	Nonsense	IPO-Porto–Portugal	EI_1104	F	62	Intestinal	Y
	NM_000051.4:c.5497-2A>C	NP_000042.3:p.?	Splice acceptor	HGC^2^G–Mexico	I_26254	M	42	Intestinal	Y
	NM_000051.4:c.5690del	NP_000042.3:p.(Phe1897SerfsTer20)	Frameshift deletion	HGC^2^G–Colombia	I_27515	M	45	Intestinal	N
	NM_000051.4:c.7681_7682del	NP_000042.3:p.(Leu2561GlyfsTer9)	Frameshift deletion	HGC^2^G–Colombia	I_9149	F	49	Mixed	N
	NM_000051.4: c.8851-1G>C	NP_000042.3:p.?	Splice acceptor	IPO-Porto–Portugal	EI_1083	F	61	Mixed	Y
	NM_000051.4:c.9079dup	NP_000042.3:p.(Ser3027LysfsTer36)	Frameshift insertion	IPO-Porto–Portugal	EI_1077	M	54	Mixed	Y
	NM_000051.4:c.9079dup	NP_000042.3:p.(Ser3027LysfsTer36)	Frameshift insertion	IPO-Porto–Portugal	EI_1143	M	60	Diffuse	Y
	NM_000051.4:c.1235+1G>A	NP_000042.3:p.?	Splice donor	INC–Colombia	EI_1005	F	44	Diffuse	Y
	NM_000051.4:c.2250G>A	NP_000042.3:p.?	Synonymous-splice	INC–Colombia	EI_1048	F	35	Diffuse	N
	NM_000051.4:c.3214G>T	NP_000042.3:p.(Glu1072Ter)	Nonsense	HGC^2^G–Honduras	I_27604	M	37	Intestinal	N
	NM_000051.4:c.5908C>T	NP_000042.3:p.(Gln1970Ter)	Nonsense	HGC^2^G–Honduras	I_27583	F	42	Intestinal	N
*BRCA2*	NM_000059.4:c.156_157insAlu	NP_000050.3:p.?	Frameshift insertion	IPO-Porto–Portugal	EI_1080	M	60	Mixed	Y
	NM_000059.4:c.2979G>A	NP_000050.3:p.(Trp993Ter)	Nonsense	INC–Colombia	EI_1061	M	80	Intestinal	Y
	NM_000059.4:c.3264dup	NP_000050.3:p.(Gln1089SerfsTer10)	Frameshift insertion	HGC^2^G–Mexico	I_2714	F	81	Intestinal	N
	NM_000059.4:c.3860del	NP_000050.3:p.(Asn1287IlefsTer6)	Frameshift deletion	HGC^2^G–Spain	I_26895	F	55	Intestinal	N
	NM_000059.4:c.4329dup	NP_000050.3:p.(Asn1444Ter)	Nonsense	HGC^2^G–Colombia	I_27118	F	75	Diffuse	Y
	NM_000059.4:c.4740_4741dup	NP_000050.3:p.(Glu1581ValfsTer37)	Frameshift insertion	HGC^2^G–Chile	I_26873	M	44	Unknown	Y
	NM_000059.4:c.7258del	NP_000050.3:p.(Glu2420AsnfsTer49)	Frameshift deletion	IPO-Porto–Portugal	EI_1139	F	76	Diffuse	Y
	NM_000059.4:c.9382C>T	NP_000050.3:p.(Arg3128Ter)	Nonsense	IPO-Porto–Portugal	EI_1137	M	40	Diffuse	N
	NM_000059.4:c.8168A>G	NP_000050.3:p.(Asp2723Gly)	Missense	HGC^2^G–Mexico	I_2757	F	45	Diffuse	N
	NM_000059.4:c.5120del	NP_000050.3:p.(Thr1707MetfsTer5)	Frameshift deletion	HGC^2^G–Honduras	I_27569	M	50	Diffuse	N
*MLH1*	NM_000249.4:c.790+1G>A	NP_000240.1:p.?	Splice donor	INC–Colombia	EI_1047	M	55	Diffuse	Y
	NM_000249.4:c.790+1G>A	NP_000240.1:p.?	Splice donor	INC–Colombia	EI_1058	M	46	Intestinal	N
	NM_000249.4:c.213_215del	NP_000240.1:p.(Glu71del)	Frameshift deletion	INC–Colombia	EI_1055	F	38	Intestinal	Y
	NM_000249.4:c.676C>T	NP_000240.1:p.(Arg226Ter)	Nonsense	HGC^2^G–Mexico	I_26265	M	48	Diffuse	N
	NM_000249.4:c.1731G>A	NP_000240.1:p.?	Synonymous-splice	HGC^2^G–Mexico	I_26197	F	46	Intestinal	Y
*PALB2*	NM_024675.4:c.2288_2291del	NP_078951.2:p.(Leu763Ter)	Nonsense	HGC^2^G–Colombia	I_12495	M	64	Intestinal	Y
	NM_024675.4:c.1240C>T	NP_078951.2:p.(Arg414Ter)	Nonsense	IPO-Porto–Portugal	EI_1109	F	62	Intestinal	Y
*TP53*	NM_000546.6:c.1010G>A	NP_000537.3:p.(Arg337His)	Missense	IPO-Porto–Portugal	EI_1106	M	62	Intestinal	Y
	NM_000546.6:c.809T>C	NP_000537.3:p.(Phe270Ser)	Missense	HGC^2^G–Mexico	I_26633	M	74	Intestinal	Y
	NM_000546.6:c.524G>A	NP_000537.3:p.(Arg175His)	Missense	HGC^2^G–Colombia	I_27525	M	46	Intestinal	Y
*RAD51D*	NM_002878.4:c.694C>T	NP_002869.3:p.(Arg232Ter)	Nonsense	IPO-Porto–Portugal	EI_1082	M	45	Mixed	Y
	NM_002878.4:c.694C>T	NP_002869.3:p.(Arg232Ter)	Nonsense	IPO-Porto–Portugal	EI_1155	F	57	Diffuse	N
*BRCA1*	NM_007294.4:c.3331_3334del	NP_009225.1:p.(Gln1111AsnfsTer5)	Frameshift deletion	HGC^2^G–Colombia	I_12491	F	43	Diffuse	N
	NM_007294.4:c.1674del	NP_009225.1:p.(Gly559ValfsTer13)	Frameshift deletion	HGC^2^G–Colombia	I_5989	M	65	Unknown	Y
*CHEK2*	NM_007194.4:c.846+1G>C	NP_009125.1:p.?	Splice donor	HGC^2^G–Colombia	I_9186	M	64	Intestinal	N
	NM_007194.4:c.349A>G	NP_009125.1:p.(Arg117Gly)	Missense	IPO-Porto–Portugal	EI_1103	F	55	Intestinal	Y
*RAD51C*	NM_058216.3:c.561_562del	NP_478123.1:p.(His187GlnfsTer15)	Frameshift deletion	INC–Colombia	EI_1035	F	45	Intestinal	N
*PMS2*	NM_000535.7:c.803+2T>G	NP_000526.2:p.?	Splice donor	IPO-Porto–Portugal	EI_1105	F	51	Intestinal	N

HGVS: Human Genome Variation Society, FH: Family history, GC: Gastric cancer, F: Female, M: Male, Y: Yes, N: No.

Recent studies have suggested the significance of P/LP variants in the *RHOA* and *CTNND1* genes.^31^, ^32^, ^33^ We did not include these genes in our primary analysis because they are not covered by the panels tested in the INC and IPO-Porto cohorts. However, we examined the 422 patients in the HGC^2^G cohort with WES data and found no P/LP variants in those genes (Supplementary Table S6). Additionally, the *CTNND1* gene was analysed in 208 diffuse and mixed gastric cancer patients from Portugal's IPO-Porto cohort, and we did not identify any pathogenic variants, as we have previously reported.^12^

**Fig. 1:**
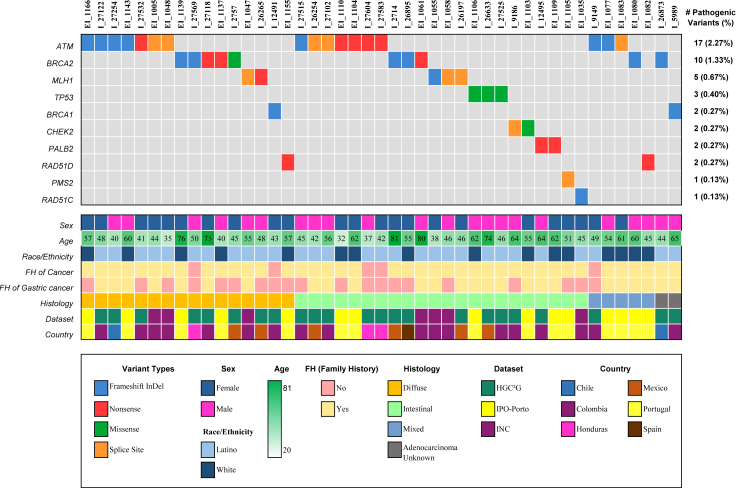
**Landscape of pathogenic and likely pathogenic germline variants identified in index patients with gastric cancer.** Characteristics of 45 patients carrying germline pathogenic or likely pathogenic variants. In the first panel, each column represents a patient, and each row a gene, colour indicates variant classification type as indicated in legend. The second panel shows the clinical characteristics of each patient, colour coded as indicated in the panel legend.

Clinical characteristics of patients who are carriers of P/LP variants are shown in Table 2, Fig. 1, and Supplementary Tables S3–S5. *ATM* was the most frequently mutated gene, with 17 patients (2.27%) carrying P/LP variants. Seven of these patients had diffuse, seven intestinal, and three mixed GC. They were diagnosed between the ages of 32 years and 62 years, and most of the patients (11 out of 17) had a family history of GC. Three of the five patients with mutated *ATM* without a family history of GC had a family history of other cancers, including breast and colon cancer. Ten patients (1.33%) were identified with a *BRCA2* P/LP variant; five were diagnosed with diffuse, three with intestinal, one with mixed GC, and one with unknown GC histology, with age of diagnosis ranging between 40 years and 81 years; five of them had a family history of GC, and four did not have a GC family history but had a family history of other cancer types, including breast and ovarian cancer. Five patients (0.67%) presented *MLH1* P/LP variants, two with diffuse and three with intestinal GC. The age of diagnosis ranged between 38 years and 55 years, and three of them had a family history of GC. Two of the patients carrying *MLH1* variants did not have GC family history but had relatives with colorectal cancer. Three patients (0.40%) had *TP53* P/LP variants, all with intestinal GC (at ages 46 years, 62 years, and 74 years) and with GC family history, and one of them also presented family history of breast and brain cancer. Two patients (0.27%) with GC family history, diagnosed with intestinal GC at ages 62 years and 64 years, harboured *PALB2* P/LP variants. *BRCA1* P/LP variants were identified in two patients (0.27%), one with a diffuse GC at 43 years and no family history of cancer and another with an unknown histology GC at 66 years and GC family history. We also identified *CHEK2* P/LP variants in two patients (0.27%); both had intestinal GC at 55 years and 64 years and a family history of breast, prostate, and pancreatic cancer. A *RAD51D* P/LP variant was identified in two patients (0.27%), one with diffuse GC at 57 years and a family history of breast cancer and another one with mixed GC at 45 years and with a GC family history. One patient was diagnosed with intestinal GC at age 45 years, with a family history of thyroid cancer and a pathogenic *RAD51C* variant (0.13%). Lastly, one patient with intestinal GC diagnosed at 51 years, with a family history of pancreatic cancer, harboured a pathogenic *PMS2* variant (0.13%).

### Genetic testing criteria for patients carrying pathogenic/likely pathogenic variants

Among the 45 patients carrying P/LP variants, we aimed to identify the cancer predisposition syndromes indicated by their results. Our assessment considered factors such as age at diagnosis, family history, the specific mutated gene, and histological type (Fig. 1, Supplementary Table S5, Supplementary Figures S1–S6). Of these 45 patients, 25 had a family history of GC. However, 15 out of the 20 patients without a family history of GC reported first and/or second-degree relatives with other cancers, including but not restricted to colorectal, breast, ovarian, endometrial, and testicular cancer. Only five patients did not report a cancer family history of cancer. Among these 45 patients, 11 out of the 16 with diffuse tumours fulfilled the HDGC criteria for testing the *CDH1*/*CTNNA1* genes, and two of these 11 also fulfilled the criteria for Lynch syndrome (both with a pathogenic variant in the *MLH1* gene). Out of five remaining carriers with diffuse GC not meeting HDGC criteria, one met Hereditary Breast and Ovarian Cancer (HBOC) syndrome testing criteria and had a *BRCA2* variant. Of the remaining 29 carriers with intestinal, mixed, or unknown GC, five fulfilled the criteria for genetic testing for known predisposition cancer syndromes. One *BRCA2* pathogenic variant carrier and one *CHEK2* LP variant carrier met the testing criteria for HBOC syndrome. Two patients carrying pathogenic variants in *TP53* fulfilled the Chompret testing criteria for Li-Fraumeni syndrome, while one carrier of a pathogenic variant in *MLH1* met the criteria for Lynch syndrome. Overall, 28 carriers, representing 62% of the cases with P/LP variants, did not qualify for genetic testing for any known cancer predisposition syndrome. As shown in Supplementary Figures S1–S5, there was evidence of co-segregation of P/LP variants in five families where relatives were tested. EI_1104 and her sister, both diagnosed with GC, carry the *ATM* p.(Arg1618Ter) variant. EI_1109 and her sister, diagnosed with GC, along with a sister who had pancreatic cancer and a brother with colon cancer, all carry the *PALB2* p.(Arg414Ter) variant. EI_1106 has a sister with GC, a sister with breast cancer, and a brother with brain cancer, all of whom carry the *TP53* p.(Arg337His) variant. EI_1103 has a sister with breast cancer and a brother with prostate cancer, both of whom carry the *CHEK2* p.(Arg117Gly) variant. While EI_1080 has three deceased relatives with GC that were not tested for variants, his brother who was diagnosed with prostate cancer carries the *BRCA2* c.156_157insAlu variant.

### Distribution of patients carrying P/LP by country of origin

When stratified by country of origin, the P/LP variant prevalence was 6.7% in Honduras, 6.5% in Mexico, 6.3% in Colombia, 6.1% in Chile, 5.8% in Portugal, and 3.4% in Spain. We did not detect P/LP variants in Uruguay, likely due to the small sample size (n = 7). We did not find differences in P/LP variant frequency between Latin American (29 of 463) and Spanish/Portuguese patients (16 of 287; *p-value* = 0.754 [Fisher exact test]). Our study included patients with early-onset GC (diagnosed by age 50 years, with or without a family history of cancer) and patients with late-onset GC (diagnosed at age >50 years) and with a family history of any cancer. The P/LP variant prevalence in patients with early onset (8.8%, 18/205) with a family history of any cancer was higher than those without a family history of cancer (2.7%, 5/187; *p-value* = 0.016 [Fisher exact test]), and in late-onset patients with a family history of any cancer (6.1%, 22/358; *p-value* = 0.306 [Fisher exact test]). The difference in P/LP variant frequency among patients diagnosed before or at/after age 50 years was not significant (23/392 vs. 22/358 carriers; *p-value* = 0.879 [Fisher exact test]), but the difference between those with a family history of cancer vs. those without was statistically significant (40/563 vs. 5/187 carriers; *p-value* = 0.0314 [Fisher exact test]). When analyses were restricted to patients with a GC family history, the P/LP variant prevalence was 1.68-fold higher in those with a GC family history (25/312, 8.01%) than in those without GC family history (18/377, 4.77%), but this difference was not significant (*p-value* = 0.084 [Fisher exact test]).

The comparison of family history between patients with P/LP variants tested via WES from population studies (HGC^2^G) and those tested through panels from hereditary cancer clinics (INC and IPO-Porto), as shown in Supplementary Table S7, reveals statistically significant differences in the prevalence of family history for any cancer (*p-value* = 0.049 [Fisher exact test]) and specifically for breast and/or ovarian cancer (*p-value* = 0.036 [Fisher exact test]). In contrast, no significant differences were observed for family history of GC (*p-value* = 0.373 [Fisher exact test]), nor for GC cases excluding those with a concurrent family history of breast and/or ovarian cancer (*p-value* = 1.00 [Fisher exact test]). These findings suggest a potential enrichment of hereditary cancer risk, particularly for breast and/or ovarian cancer, in the hereditary cancer clinic cohorts from INC and IPO-Porto.

### Association testing of P/LP variants and GC

We compared the frequency of P/LP variants between our sample population and gnomAD (v4.1) to assess the likelihood of finding these variants by chance (Supplementary Table S8). We found that P/LP in *ATM*, *BRCA2*, *MLH1*, *RAD51D*, and *TP53* were more prevalent in our GC cases, supporting a causal role for variants in these genes. In contrast, despite the frequency of P/LP variants in *BRCA1*, *CHEK2*, *PALB2*, *PMS2*, and *RAD51C* in our cases being slightly higher than gnomAD, the associations were not statistically significant. Sample size is limited in our study; however, we are reporting the prevalence of these gene variants in patients with GC from Latin American and European ancestry, and that is consistent with results from other studies that show an association with GC, including *BRCA1*,^34^ *CHEK2*,^35^ *PMS2*,^36^ *RAD51D*,^37^^,^^38^ *PALB2*^13^^,^^34^ and *RAD51C*.^13^^,^^34^ These genes play crucial roles in essential biological processes, such as DNA repair, cell replication, and apoptosis. Additionally, several studies have indicated a causal relationship between homologous recombination genes and predisposition to GC.^13^, ^14^, ^15^^,^^39^ Also, it is important to note that phenotype data is generally unavailable for samples in gnomAD, and the database is not curated by diagnosis or family history of cancer.

### Tumour histology, *Helicobacter pylori* infection, age, and sex distribution in patients carrying P/LP variant(s)

When stratified by tumour histology, P/LP variant prevalence was >2-fold significantly higher in patients with intestinal tumours (9.8%, 22/224) compared to diffuse tumours (4.3%, 16/376; *p-value* = 0.007 [Pearson's Chi-square]) and not significant when compared to mixed tumours (4.5%, 5/110; *p-value* = 0.096 [Pearson's Chi-square]). Out of 750 cases, we had data on *Helicobacter pylori* infection for 233 cases. Of these, 74.4% tested positive for the infection. In contrast with a previous report,^34^^,^^40^ we failed to detect a significant difference in infection rates between patients carrying P/LP variant (11 out 17, 64.7%) and non-carriers (155 out 206, 75.2%; *p-value* = 0.386 [Fisher exact test], Supplementary Table S9). The average age of diagnosis among non-carriers was age 51.3 years (range: 19 years–86 years), with no significant differences noted when compared with carriers (52.8 years; range: 32 years–81 years; *p-value* = 0.154 [F-test]). Also, there was no significant difference in age of diagnosis among patient carriers stratified by histology (diffuse: 50.9 years; mixed: 53.8 years; intestinal: 53.9 years; *p-value* > 0.1 [F-test] for all comparisons) or sex (women: 52.2 years vs. men: 53.5 years; *p-value* = 0.293 [F-test]).

### Tumour analysis of gastric carcinomas of patient carrying P/LP variants

**Fig. 2:**
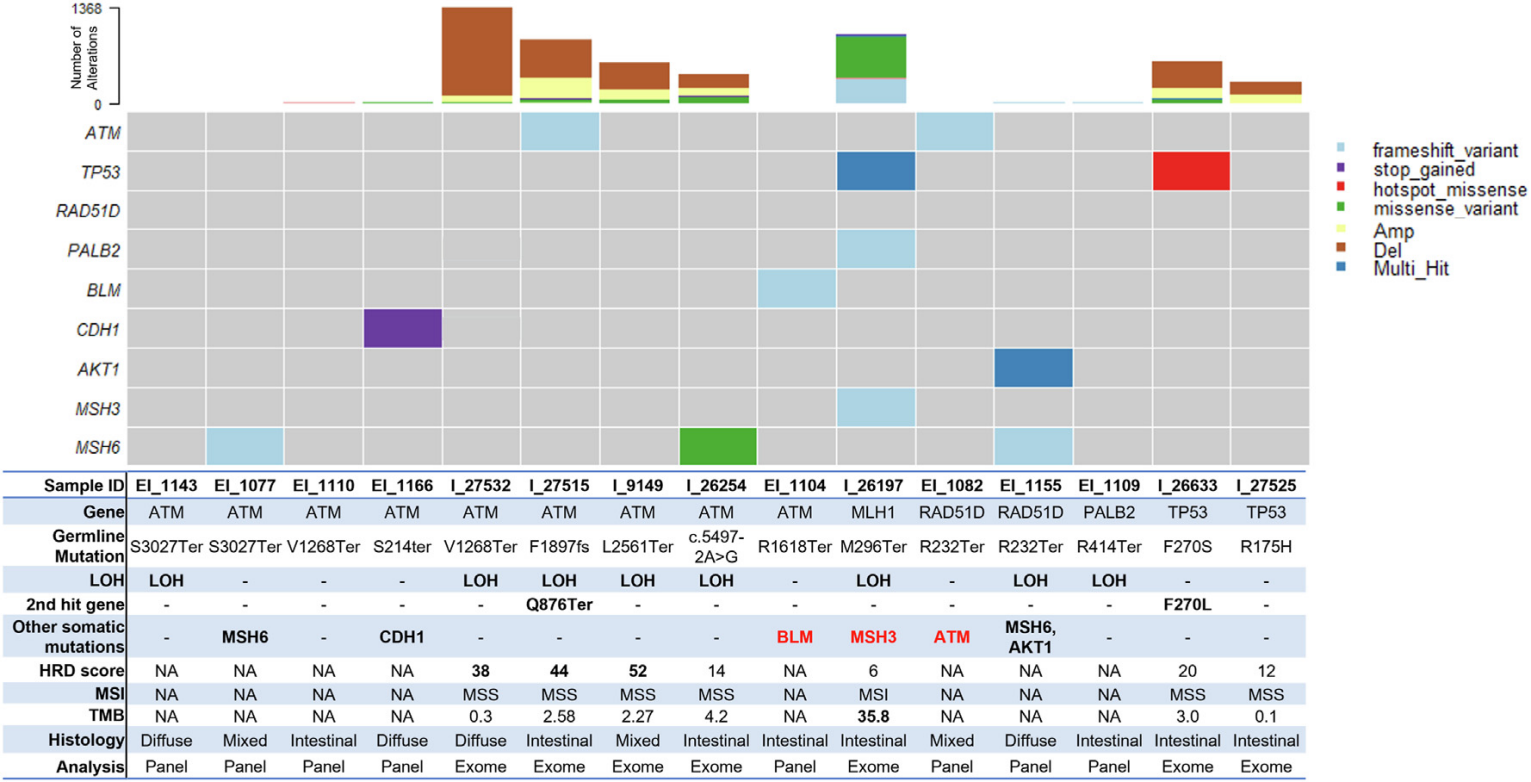
**Somatic analysis of patients with gastric cancer, carriers of pathogenic germline variants.** Oncoplot depicting somatic mutations from patients with P/LP germline variant in which somatic data available by whole exome (n = 7), or targeted panel (n = 8). Each column represents a patient and each row a gene, colour indicates variant classification type as indicated in legend. The table below indicates detailed patient and mutation information. Genes in red represent mutations in a gene within the same pathway as the initial germline variation. Homologous recombination deficiency (HRD) score calculated based on loss of heterozygosity (LOH), telomeric allelic imbalance, and large-scale state transition for whole exome data only. Microsatellite instability (MSI) score was calculated using MSISensor-Pro for whole exome data only and confirmed by analysing the fragment length variations of five markers within the “Bethesda panel”. Tumour Mutation Burden (TMB) was calculated for exome data only as number of somatic mutations per megabase.

We performed panel testing in tumour samples from Portugal (n = 8) and WES in tumour samples from Colombia (n = 4) and Mexico (n = 3) in carriers of P/LP variants in *ATM* (nine cases), *TP53* (two cases), *RAD51D* (two cases), *PALB2* (one case), and *MLH1* (one case) (Fig. 2, Supplementary Table S10). The NGS-based tumour test detected all germline variants, and the variant allele frequency (VAF) ranged between 4% and 81%. Eight of the 15 tumours had evidence of loss of heterozygosity (LOH) defined as having a purity-adjusted VAF greater than 70%, suggesting a biallelic inactivation by deletion of the wild-type allele. The tumour from patient I_26197, a carrier of an *MLH1* germline variant, exhibited microsatellite instability, a higher number of missense variants, and had a mutation in *MSH3*, which also belongs to the mismatch repair pathway (MMR). Two of the tumours showed a second hit within the same gene, potentially further exacerbating the effects of the germline variation. In particular, the tumour from patient I_26623 showed a second hit in the same amino acid *TP53* F270L, found only in reads that do not contain the germline variant. Four of the tumours had P/LP somatic mutations in genes within the same pathway as the germline variants, further affecting the functionality of that repair process. Three of the tumours showed P/LP variants in other known gastric cancer driver genes. The homologous recombination deficiency (HRD) score was determined for the WES samples. Four carriers of *ATM* variants presented the highest HRD scores (>38), of the seven samples tested. For case EI_1109, we identified a second relative with GC who carried the same *PALB2* variant (Arg414Ter, Supplementary Figure S4) and showed loss of heterozygosity (LOH) in her gastric tumour (Fig. 2). Overall, these data provide evidence suggesting causality of the germline variants identified in our study.

## Discussion

This study evaluated the frequency of P/LP germline variants in known multi-tumour, moderate-to-high penetrance cancer predisposition genes on the risk of GC, regardless of family history, age of onset, or the histological subtype. Overall, 45 (6%) out of 750 patients carried P/LP variants in known cancer predisposition genes (*ATM*, *BRCA1*, *BRCA2*, *PALB2*, *TP53*, *RAD51D*, *RAD51C*, *CHEK2*, *MLH1*, and *PMS2*. Table 2, Fig. 1). All these genes play a crucial role in DNA damage repair machinery. Namely, homologous recombination genes have been associated with GC predisposition in either selected^8^^,^^11^^,^^12^^,^^14^, ^15^, ^16^ or unselected GC cohorts.^34^^,^^41^, ^42^, ^43^, ^44^ The reported prevalence of P/LP variants in these cancer genes in patients with GC ranges between 3% and 11%.^13^^,^^15^^,^^39^^,^^45^ Here, we report a prevalence of 6%, consistent with previous reports. Notably, in our cohort, patients with P/LP variants display heterogeneous histology, including intestinal, mixed, and diffuse GC. When stratified by tumour histology, the prevalence of P/LP variants was higher in carriers diagnosed with intestinal GC (9.8%) than those with diffuse and mixed GC (4.3% and 4.7%, respectively). This was consistent with the fact that most previous GC genetic studies focused on patients and families with diffuse GC tumours and/or fulfilling HDGC criteria. Hence, our study shows that many patients with intestinal GC tumours, who based on current guidelines are not recommended genetic testing, can also carry P/LP changes. Additionally, the testing criteria for the intestinal histological type are not very specific or well-defined.^46^ Therefore, when we studied these cases, which were deliberately selected to have all three GC histology subtypes (not just diffuse tumours, as most previous studies), and using a panel of known cancer predisposition genes, the probability of identifying a P/LP variant in a GC case, of any histology, was significantly increased.

P/LP variant prevalence was higher in the group of patients diagnosed before age 50 years with a family history of any cancer and in the group with a family history regardless of the age of onset (8.8% and 6.1%, respectively) than in the group of patients diagnosed before age 50 years without a family history (2.7%). Among the 45 patients with a P/LP variant, 28 did not fulfil genetic testing criteria for any known cancer predisposition syndrome, and 17 met the criteria for genetic testing for specific hereditary cancer syndromes, such as HDGC, Lynch, HBOC, or Li-Fraumeni syndromes. However, of the 17 patients who fulfilled the criteria for genetic testing for known cancer predisposition syndromes, only five would have been correctly identified if a comprehensive gene panel had not been analysed. Therefore, regardless of GC histology subtype, our findings suggest testing should be carried out in patients with a family history of any cancer, irrespective of the age of diagnosis, using a panel of known cancer predisposition genes.

In our study, the average age of diagnosis of the patients carrying P/LP variants was 52.8 years, which is very similar to the average age of non-carrier diagnosis (51.3 years). These similarities can be explained by the moderate penetrance of the genes studied, which lead to a later onset of the disease compared to established syndromes like HDGC, where the average age of diagnosis is ∼40 years.^47^^,^^48^ This difference in age at diagnosis has been reported in the literature.^34^^,^^45^ Patients with *CDH1* pathogenic variants tend to be diagnosed at a younger age compared to non-carriers. In contrast, the age at diagnosis for individuals with a pathogenic variant in the other known cancer predisposition genes was similar to that of non-carriers.^34^^,^^45^ Notably, our observations are consistent with a previous study indicating that individuals from FIGC families develop intestinal GC at a higher average age than those with HDGC.^46^ Moreover, our cohort was selected based on early-onset GC and/or family history, so the families negative for pathogenic variants in the genes studied can have a predisposition genetic cause not yet identified.

When designing our study, in addition to including patients with all histological types, we deliberately included patients with a family history of any cancer because: i) overall cancer family history is relatively more straightforward to obtain; ii) GC family history is rare, particularly in low-incidence countries; iii) GC is a late onset malignancy and competing mortality may limit the accurate obtention of GC-specific family history^49^; iv) our own studies and those from others have suggested multi-organ genes likely increased the risk of GC.^8^, ^9^, ^10^, ^11^, ^12^, ^13^, ^14^, ^15^, ^16^ Casting this ‘wider net’, therefore, was a strength of our study, and it allowed us to conclude that using two relatively more straightforward types of information, such as the age of onset and cancer family history, would allow genetic counsellors and clinicians to identify patients with GC, who likely carry germline P/LP variants in these genes. Although we acknowledge that more studies need to be carried out to refine further the GC risk associated with variants in these genes, our study suggests that the GC risk assessment field should shift from the paradigm of focussing primarily on patients with diffuse tumours or HDGC family history to one that considers early onset, all histology subtypes and family history of any cancer.

The criteria for including patients in our study were based on the age at diagnosis or family history of any cancer and the requirement that the index case had GC. This was true regardless of any specific types of other cancers present in the family. Additionally, patients fulfilling clinical criteria for HDGC were tested for germline P/LP variants in *CDH1* or *CTNNA1*, and P/LP variant-positive patients were excluded. Even though the recruitment criteria did not specifically target cases with a family history of breast and ovarian cancer, these cancers were the most frequently reported in the relatives of our index patients. This prevalence may be linked to syndromes and P/LP variants in genes such as *ATM*, *BRCA1*, *BRCA2*, and *CHEK2*, which are known to predispose individuals to GC, as noted in other studies.^13^, ^14^, ^15^^,^^34^^,^^39^ While we recognise the possibility that these P/LP variants may be linked to other cancers (e.g., those seen in HBOC), in the few families where we performed co-segregation analyses, there were multiple GC cases within the family carrying the P/LP variant, providing further GC causality evidence for our findings.

A significant strength of our study was our evaluation of unselected patients from case-control studies (HGC^2^G) and hereditary cancer clinics (INC and IPO-Porto). We did not find major differences between patients recruited by the HGC^2^G Consortium and those from hereditary cancer clinics. However, we acknowledge that the age of diagnosis was significantly lower in the hereditary cancer clinics, suggesting a possible bias in identifying more P/LP variants and families with HBOC in these clinics (Supplementary Table S7, which shows that a higher fraction of patients from clinics have family history of breast and ovarian cancer), as patients were referred for genetic cancer risk assessment due to their early diagnosis and family history of cancer. Furthermore, since patients recruited by the hereditary cancer clinics were sequenced using panels with better coverage of the genes of interest, some positive cases in the HGC^2^G cohort may have been missed due to the use of whole exome sequencing, which had lower coverage than the panels for the genes of interest. Therefore, while we observed an association between these P/LP variants and GC in participants from hereditary clinics, we cannot rule out the possibility that these variants are linked to cancers other than GCs. Nevertheless, the evidence of P/LP variants co-segregation and the enrichment in unselected patients (See Supplementary Figure S7, with half of our P/LP variant carriers recruited by the HGC^2^G Consortium) supports their association with GC.

### Caveats and limitations

Our study has several limitations. First, our study lacks details on *H. pylori* infection in most cases, which is an important risk factor, especially in Latin America.^13^^,^^37^^,^^50^, ^51^, ^52^, ^53^, ^54^, ^55^, ^56^ Second, our study did not estimate the prevalence of P/LP variants in incident and unselected patients with GC. Several studies have shown that the true prevalence of P/LP variants in cancer genes in patients with GC ranges from 5 to 10%. Should this be confirmed in future and well-designed studies, given about a million patients are diagnosed with GC globally each year, the implications for prevention would be enormous, as potentially 50,000–100,000 patients each year could develop GC due to a genetic predisposition. A third limitation of our study was the lack of systematic HRD tumour testing for carriers of germline P/LP variants in HR genes. This was primarily due to our limited access to tumours from such carriers, as we only had a handful of archival blocks. However, WES analyses found some evidence of high HRD scores in tumours from *ATM* germline P/LP variant carriers (see *Tumour analyses of gastric carcinomas of patients carrying P/LP variants*, above). Finally, two additional and related limitations of our study were our limited evidence for GC causality, as association testing resulted in nominally significant values for a handful of genes (*ATM*, *BRCA2*, *MLH1*, *RAD51D*, and *TP53*; see Supplementary Table S8), and the possibility that these variants may be associated with other cancer types that are more common in HBOC or Lynch Syndrome. While co-segregation analyses and association testing in our study provided some preliminary evidence of GC causality, more systematic and extensive studies that include systematic HRD testing of gastric tumours are needed.

Despite these limitations, this study included a large, international, and multi-centre cohort with an extensive case series enriched by Latinos. Under-represented in GC research efforts,^57^^,^^58^ Latinos are a population with a distinctive demographic (shaped by an ancestral admixture of Indigenous Americans, Europeans, and Africans^59^, ^60^, ^61^, ^62^) who have a high GC burden, as well as unique GC genomic and epidemiological patterns.^4^ Our study showed that known multi-tumour, moderate-to-high penetrance cancer predisposition genes could explain a significant proportion of patients with GC, regardless of the histological type. The potential association of homologous recombination genes with GC predisposition may be clinically relevant for both cancer prevention and targeted therapies (i.e., use of PARP inhibitors).^19^, ^20^, ^21^ Likewise, patients with P/LP MMR germline variants can also benefit from PD-1 inhibitors.^63^

In conclusion, our results show that, in addition to *CDH1* and *CTNNA1* testing in families fulfilling the HDGC criteria, multi-organ cancer predisposition genes should be included in gene panels used for investigating germline variants in patients with diffuse and non-diffuse GC. We anticipate two potential applications of our findings: first, HDGC-like patients who test negative for *CDH1* and *CTNNA1* pathogenic variants, who by HDGC definition only have diffuse tumours, should be offered a broader cancer panel. Second, a broader panel could be considered for patients who do not fulfil HDGC criteria but who may have themselves and their relatives histologically heterogeneous GC (i.e., reporting diffuse and no-diffuse tumours) and/or who report relatives with cancer types overlapping with other cancer predisposition syndromes. Finally, our study failed to replicate a risk interaction between *H. pylori* infection and HR gene variants recently reported in Japan.^34^ Even though our study was carried out in a small number of patients recruited in areas with high *H. pylori* infection rates, our results suggest that further studies in non-Japanese populations should be carried out to investigate interactions between *H. pylori* infection and inherited cancer gene risk variants.

## Contributors

JG, APE-F, PCL, MMEP, MHB, JT, MRT, and LGC-C conceptualised and designed the study. MMEP, RP-M, DRM, MCS-S, MHB, JT, MRT, and LGC-C acquired funding. AHC, ADV, JEC, NFF, LC, NR-S, RM, JS, MMEP, JR-M, RP-M, JS-O, RD, GM, AMC-R, MCS-S, MHB, JT, MRT, and LGC-C collected samples and data. APE-F, JS-O, HZ, GMP-E, FC-V, GM, ALR-H, LMG-C, JEC, and RD processed samples. AHC, AM, RP-M, ARI, FS, and MHB reviewed all available tumours blocks and pathology reports. JG, CP, PP, AP, CS, AB, ALR-H, JR-M, and performed panel testing. PCL, KAC, and DJM curated data and developed the pipeline for WES. JG, APE-F, ALR-H, and LMG-C validated the variants. APE-F, PCL, and LGC-C had access to all the data. JG, APE-F, PCL, and PP completed the data analysis. All authors reviewed and interpreted the data. JG and APE-F drafted the article, and all authors reviewed and edited it. DRM, MCS-S, MHB, JT, MRT, and LGC-C supervised the study. The Hispanic Gastric Cancer Genetics Collaborative Group (HGC^2^G) and the Consorcio Galicia conducted patient recruitment and clinical assessment. All authors reviewed and approved the final manuscript version, assuming final responsibility for the decision to submit it for publication.

## Data sharing statement

The data presented here, obtained under multiple countries and regulations, will be available upon request to the corresponding author. Data obtained using NIH funding is available in dbGAP (Study Accession: phs003251.v1.p1). No additional data other than what was presented in the manuscript will be made available to others.

## Declaration of interests

Manuela Pinheiro is a research fellow of the Liga Portugues Contra o Cancro. None of the other authors, Consortia and groups listed have received funding or have other competing interests to declare related to this project.

## Appendix A Supplementary data

Supplementary Figures S1–S6

Consortium Information
