# Supplementary material for: The role of multi-organ cancer predisposition genes in the risk of inherited and histologically diverse gastric cancer
Source: eBioMedicine. 2025 May 29;116:105759. doi: 10.1016/j.ebiom.2025.105759 (PMC12166715; doi:10.1016/j.ebiom.2025.105759)
Supplement: Supplementary Figures S1–S6 [file mmc2.docx]

**Supplementary Figure 1.** Pedigrees of individuals carrying *ATM* P/PL variants (A. EI_1083 [c.8851-1G>C], B. EI_1104 [p.Arg1618Ter]).

**
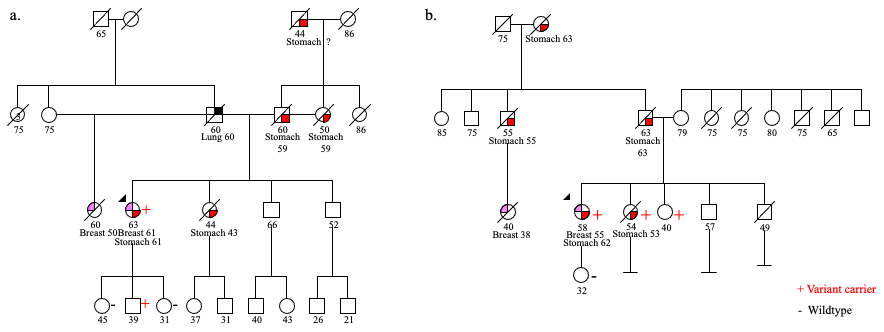
**

**Supplementary Figure 2.** Pedigree of EI_1080, a carrier of the variant *BRCA2* c.156_157insAlu.


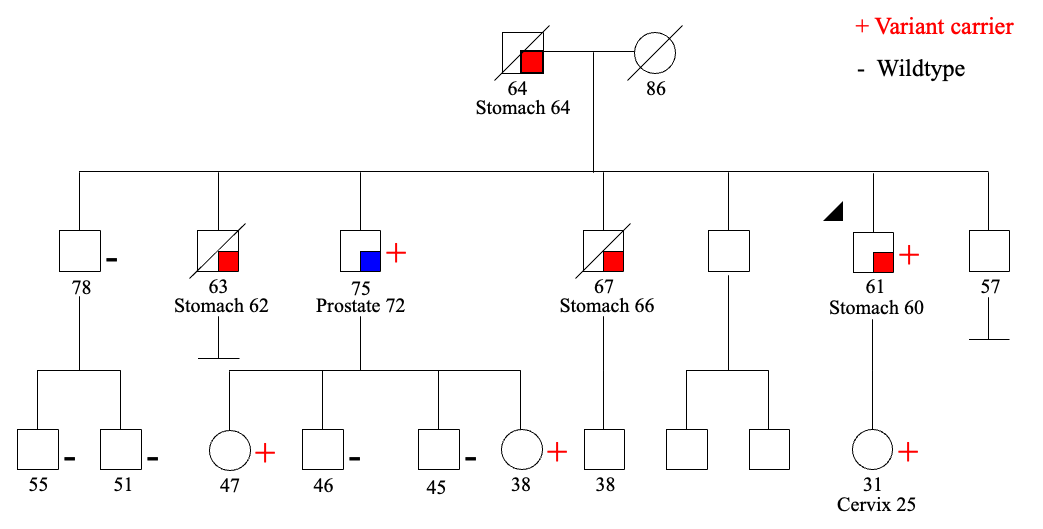


**Supplementary Figure 3.** Pedigree of EI_1103, a carrier of the variant *CHEK2* p.(Arg117Gly).


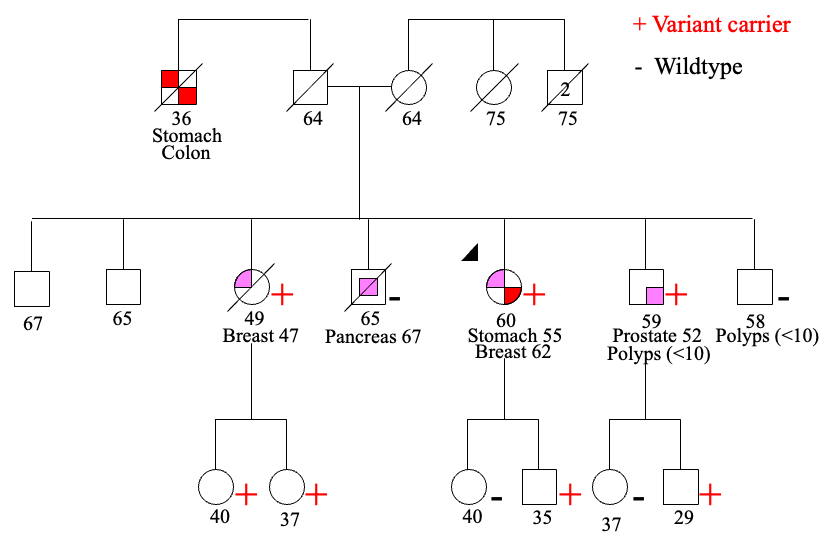


**Supplementary Figure 4.** Pedigrees of EI_1109, a carrier of the variant *PALB2* p.(Arg414Ter).


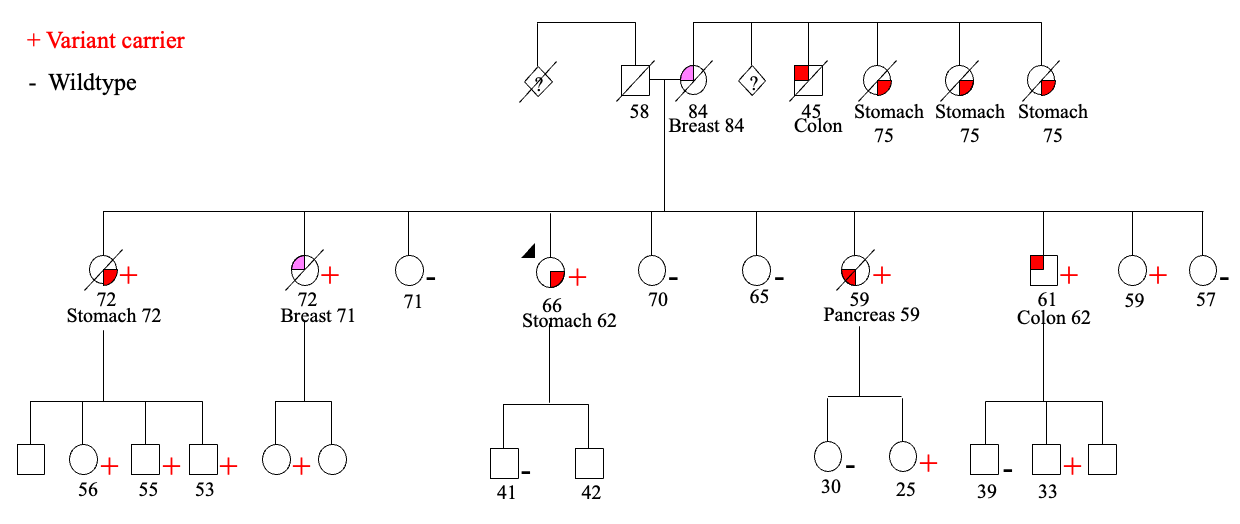


**Supplementary Figure 5.** Pedigrees of EI_1106, a carrier of the variant *TP53* p.(Arg337His)


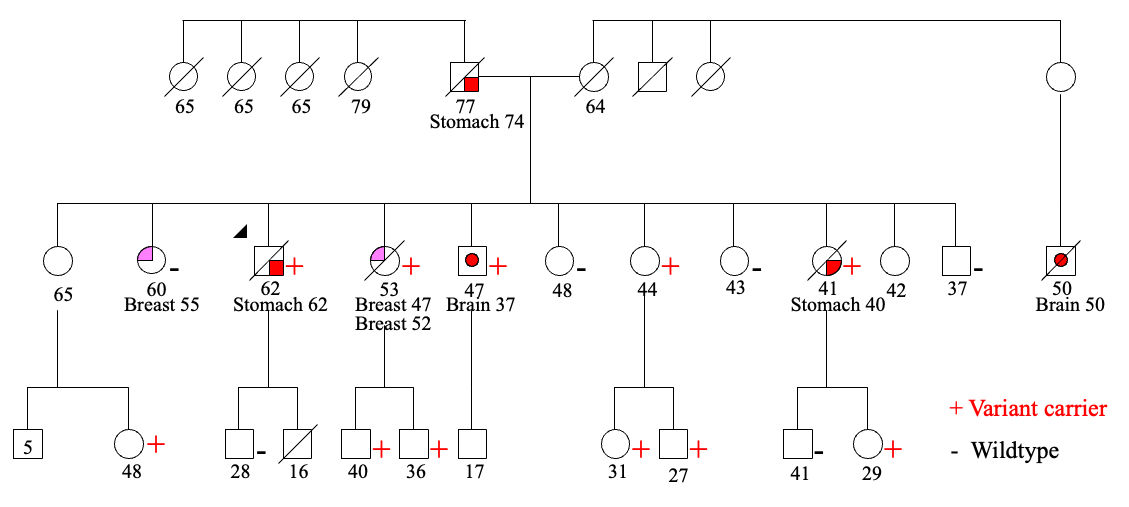


**Supplementary Figure 6.** Family trees of P/PL variant carriers without tested relatives.
