# Supplementary material for: The role of multi-organ cancer predisposition genes in the risk of inherited and histologically diverse gastric cancer
Source: eBioMedicine. 2025 May 29;116:105759. doi: 10.1016/j.ebiom.2025.105759 (PMC12166715; doi:10.1016/j.ebiom.2025.105759)
Supplement: Consortium Information [file mmc3.docx]

**Hispanic Gastric Cancer Genetics Collaborative Group (HGC^2^G):** González Patricio1, Parra Carol2, Torres Osvaldo3, Adelsdorfer Cedric4, Martínez Jose5, Norwood Dalton6, Montalvan-Sanchez Eleazar7, Nefa Florencia8, Sanchez Gloria9, Castaño Rodrigo10,11,12, García Francisco13, Buitrago Diego Andrés14.

1. Instituto de Ciencias Biomédicas, Facultad de Medicina, Universidad de Chile, Santiago, Chile

2. Laboratorio de Investigación en Nutrición y Alimentos, Departamento de Salud, Comunidad y Gesttión, Facultad de Ciencias de la Salud, Universidad de Playa Ancha, Valparaíso, Chile

3. Servicio de Cirugía Digestiva, Hospital Clínico Regional de Concepción Dr. Guillermo Grant Benavente, Concepción, Chile

4. Servicio de Cirugía, Hospital Dr. Gustavo Fricke de Viña del Mar, Viña del Mar, Chile

5. Servicio de Cirugía, Hospital Dr. Eduardo Pereira Ramírez, Valparaíso, Chile

6. Division of Preventive Medicine, Department of Medicine, The University of Alabama at Birmingham, Birmingham, AL, U.S.

7. Department of Medicine, Indiana University School of Medicine, Indianapolis.

8. Laboratorio Genia, Montevideo, Uruguay.

9. Facultad de Medicina, Universidad de Antioquia, Medellín, Colombia.

10. Grupo de Gastrohepatología, Universidad de Antioquia, Medellín, Colombia

11. Instituto de Cancerología Las Américas Auna, Medellín, Colombia

12. Departamento de Endoscopia, Clínica El Rosario Tesoro, Medellín, Colombia

13. Hospital Federico Lleras Acosta, Ibagué, Tolima

14. Clínica Internacional de Alta Tecnología, CLINALTEC, Ibagué, Colombia

**Consorcio Galicia:** Hernandez Vicent1,2, Alonso Sara1,2, Galovart Miguel1,2, Rodriguez-D'Jesus Antonio1,2, de Castro Luisa1,2, Rodriguez-Prada José Ignacio1,2, Gago-Domínguez Manuela3, Redondo-Marey Carmen4, Miranda Ponte Sara4

1. Department of Gastroenterology. Complexo Hospitalario Universitario de Vigo (CHUVI), SERGAS, Vigo, Spain

2. Research Group in Digestive Diseases, Galicia Sur Health Research Institute (IIS Galicia Sur), SERGAS-UVIGO, Vigo, Spain

3. Cancer Genetics and Epidemiology Group, Genomic Medicine Group, Instituto de Investigación Sanitaria de Santiago de Compostela (IDIS), Fundación Pública Galega Instituto de Investigación Sanitaria de Santiago de Compostela, SERGAS, Santiago de Compostela, 15706, Spain

4. Oncology and Genetics Unit, Instituto de Investigacion Sanitaria Galicia Sur (IISGS), Xerencia de Xestion Integrada de Vigo-SERGAS, Vigo 36312, Spain.
